# Mechanistic optimization of inavolisib combined with CDK4/6 inhibitors in the treatment of PIK3CA-mutated breast tumors

**DOI:** 10.3389/fimmu.2025.1693927

**Published:** 2025-11-06

**Authors:** Rongyu Zhu, Haixin Zhang, Fuli Zhang

**Affiliations:** 1 School of Graduate Students, Heilongjiang University of Chinese Medicine, Harbin, Heilongjiang, China; 2 Department of Warm Disease Teaching and Research Section, Heilongjiang University of Chinese Medicine, Harbin, Heilongjiang, China

**Keywords:** inavolisib, CDK4/6 inhibitor, PI3Kα pathway, PIK3CA mutation, synergistic mechanism

## Abstract

PIK3CA mutations are common oncogenic mutations in breast cancer, and abnormal activation of the PI3K/AKT/mTOR pathway is a key mechanism underlying tumorigenesis and drug resistance. Inavolisib is a selective PI3Kα inhibitor approved for the treatment of hormone receptor-positive breast cancer with PIK3CA mutations. CDK4/6 inhibitors (such as palbociclib and ribociclib) block the transition from the G1 to S phase of the cell cycle and have become standard treatment for hormone receptor-positive breast cancer. Both agents exhibit resistance issues when used as monotherapy, particularly in the context of PIK3CA mutations. Studies have shown that the combination of CDK4/6 inhibitors with PI3K inhibitors (such as inavolisib) significantly enhances antitumor efficacy. Additionally, the combination therapy effectively inhibits tumor cell proliferation and induces apoptosis. In preclinical studies, this combination strategy demonstrated significant antitumor activity in various PIK3CA-mutated xenograft models. Although clinical trials (e.g., NCT04191499) are exploring the potential of inavolisib combined with CDK4/6 inhibitors, challenges remain, including toxicity management, biomarker selection, and optimizing dosing regimens to enhance efficacy and reduce side effects. This review synthesizes preclinical and clinical evidence on the mechanistic optimization of inavolisib combined with CDK4/6 inhibitors for PIK3CA-mutated breast cancer. It covers molecular mechanisms, synergistic effects, resistance strategies, biomarkers, and future directions, with an emphasis on immunological implications. The scope is limited to HR+/HER2-negative subtypes, excluding other cancers or non-PI3K-targeted therapies, to provide a focused foundation for translational immunology in oncology.

## 1 Introduction

Breast cancer is one of the most common malignant tumors in women worldwide, with the hormone receptor-positive (HR+)/human epidermal growth factor receptor 2-negative (HER2−) subtype accounting for approximately 70% of cases (1). Although endocrine therapy (ET) combined with cyclin-dependent kinase 4/6 (CDK4/6) inhibitors has significantly improved progression-free survival (PFS) in such patients (2), the presence of PIK3CA gene mutations often leads to abnormal activation of the PI3K/AKT/mTOR signaling pathway, which has become an important mechanism of endocrine resistance (3, 4).

PIK3CA gene mutations are one of the most common oncogenic mutations in breast cancer, accounting for approximately 30% of all breast cancer cases (5). PIK3CA mutations lead to the sustained activation of the PI3K/AKT/mTOR signaling pathway, thereby promoting tumor cell proliferation, survival, and invasion (6). Although PI3K inhibitors have been approved for the treatment of PIK3CA-mutated HR+/HER2- breast cancer, their monotherapy efficacy is limited, and resistance issues exist (7). Studies have shown that the combination of CDK4/6 inhibitors and PI3K inhibitors significantly enhances antitumor activity and overcomes resistance (8). For example, inavolisib (GDC-0077), a selective PI3Kα inhibitor, exhibits stronger cytotoxicity in PIK3CA-mutated breast cancer and demonstrates good antitumor effects in animal models (9). Additionally, clinical and preclinical data indicate that inavolisib combined with CDK4/6 inhibitors significantly improves antitumor efficacy, partly by coordinated suppression of cell-cycle (pRB-E2F) and mTORC1/2 signaling (9, 10).

However, the optimization of the combination therapy mechanism still requires further exploration. Studies have shown that tumors with PIK3CA mutations exhibit heterogeneity, and some subclones may develop resistance through p21-mediated DNA damage repair or activation of the PDK1 signaling bypass (11). Additionally, the timing of administration, dose adjustment, and biomarker selection (such as PIK3CA mutation abundance and ESR1 co-mutations) between CDK4/6 inhibitors and PI3K inhibitors may influence treatment efficacy (12, 13). For example, circulating tumor DNA (ctDNA) analysis revealed that patients with multiple mutations at baseline had significantly higher overall response rates (ORR) than those with single mutations, suggesting that the mutation burden may serve as a predictive marker for efficacy (14, 15). These findings provide a theoretical basis for the precision optimization of combination regimens.

In summary, the combination of Inavolisib and CDK4/6 inhibitors provides a new treatment paradigm for PIK3CA-mutated breast cancer by synergistically blocking the PI3K signaling pathway and cell cycle progression. This study aims to systematically elucidate the scientific basis for mechanism optimization, integrate preclinical model data with clinical trial results, explore strategies for overcoming drug resistance, and explore biomarker-guided personalized treatment directions, with the goal of providing theoretical support for clinical practice.

## 2 Mechanism of action of inavolisib

**Figure 1:**
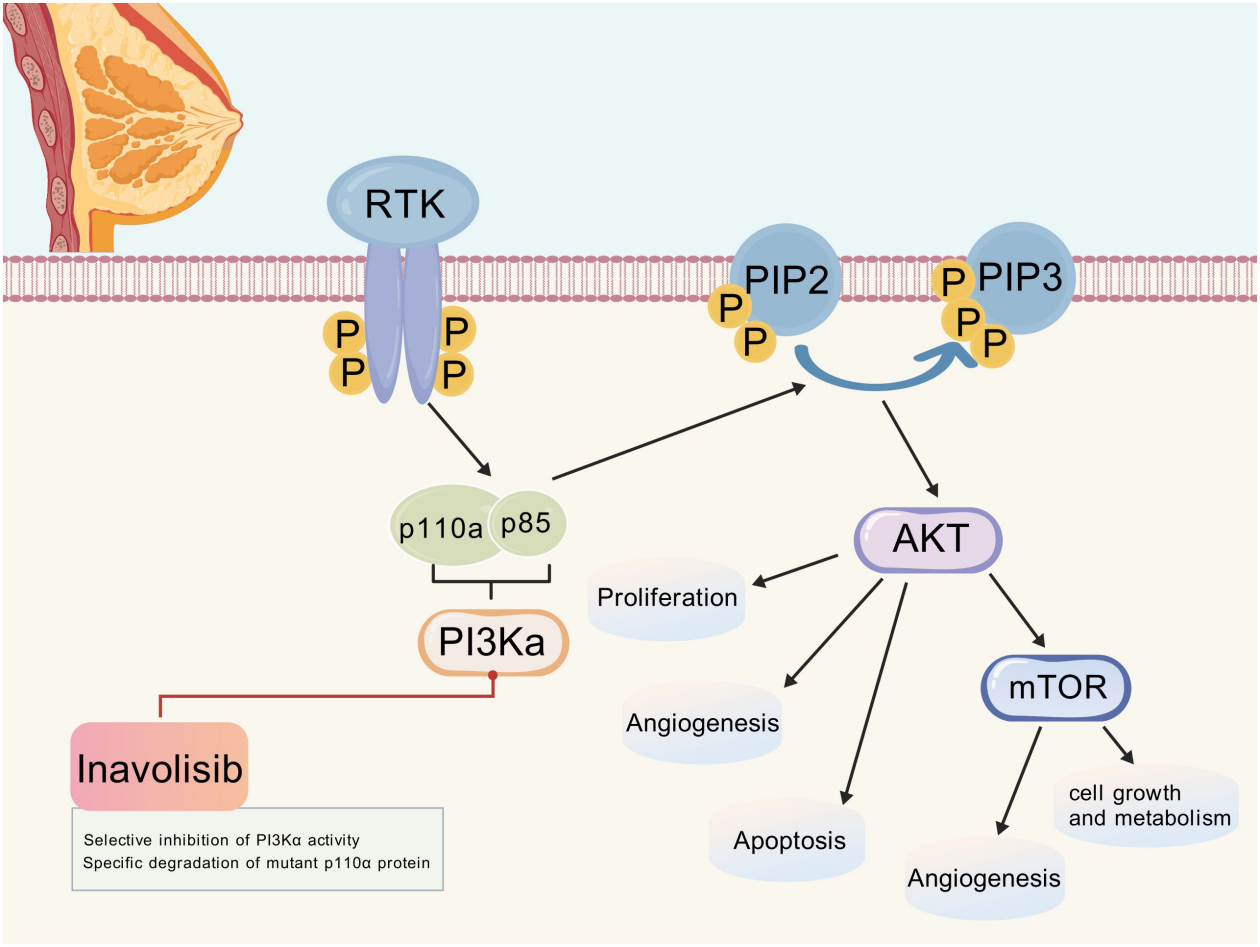
PI3K/AKT/mTOR signaling pathway and the mechanism of action of inavolisib. (Created with BioGDP.com). After RTK activation, PI3Kα is recruited and activated, which converts PIP2 to PIP3. PIP3 then activates AKT, which in turn regulates downstream molecules such as mTOR, affecting cell proliferation, angiogenesis, apoptosis, and cellular growth and metabolism. Inavolisib selectively inhibits PI3Kα activity and can also specifically degrade mutant p110α protein, thereby interfering with the abnormal activation of this pathway. RTK, Receptor tyrosine kinase; PI3Kα, Phosphatidylinositol-3 kinase α; PIP2, Phosphatidylinositol-4,5-bisphosphate; PIP3, Phosphatidylinositol-3,4,5-trisphosphate; AKT, Protein kinase B; mTOR, Mammalian target of rapamycin.

Inavolisib (GDC-0077) is a third-generation highly selective PI3Kα inhibitor that achieves precise targeting of PIK3CA-mutated tumors through a dual-action mechanism: selectively inhibiting PI3Kα activity and inducing the degradation of mutant p110α protein (16). This mechanism not only overcomes the negative feedback activation of the PI3K pathway but also provides a new treatment paradigm for PIK3CA-mutated breast cancer by targeting the degradation of oncogenic mutant proteins (**Figure 1**).

### 2.1 Selective inhibition of PI3Kα activity

Inavolisib is a highly PI3Kα-selective compound with >300-fold reduced *in vitro* potency against PI3Kβ/δ/γ relative to PI3Kα and demonstrates preferential binding affinity for common oncogenic p110α mutants over wild-type protein (17). Structural biology studies indicate that this selectivity stems from its unique molecular characteristics. The pyridine-pyrimidine backbone deeply embeds into the ATP-binding pocket of PI3Kα, blocking the binding of the substrate PIP2. Additionally, the difluoromethyl oxazoline group forms hydrogen bond interactions with the hinge region Val828, enhancing binding stability (18). Finally, the amide side chain interacts with the E545 mutation site in the helix domain in a conformation-specific manner, preferentially recognizing the mutant active state (19). For example, preclinical data show that its inhibitory activity against the H1047R mutant (IC50 = 0.038 nM) is significantly different from that against the wild-type (IC50 not specified, but estimated to exceed 11.4 nM based on subtype selectivity) (9). By targeting and inhibiting PI3Kα, Inavolisib effectively blocks the activation of the AKT/mTOR signaling pathway, inhibiting the conversion of PIP2 to PIP3 by the PI3K catalytic subunit, thereby disrupting the membrane localization and activation of downstream effector molecules (17).

### 2.2 Specific degradation of mutant p110α protein

Inavolisib achieves selective degradation of mutant p110α through a coordinated set of molecular events. Structural and molecular dynamics analyses indicate that hotspot kinase-domain mutations (e.g., H1047R) increase conformational flexibility of the C-terminal and kinase domains, which exposes lysine residues (notably Lys495 and Lys802) that are otherwise partially buried in the wild-type conformation (9). Binding of inavolisib stabilizes an open kinase-domain conformation in the mutant protein, further increasing solvent accessibility of these lysines and creating structural epitopes favorable for ubiquitin conjugation (20, 21). Concomitantly, inhibition of PI3Kα activity can relieve negative feedback on RTKs such as HER2/HER3, altering the subcellular localization and membrane recruitment dynamics of the p85–p110 complex and promoting interaction with specific E3 ubiquitin ligases (for example, NEDD4L has been implicated in preclinical studies) (22, 23). The recruited E3 ligase catalyzes polyubiquitination of exposed lysines on mutant p110α, marking the protein for recognition and degradation by the 26S proteasome; this degradation is blocked by proteasome inhibitors such as MG132, confirming proteasome dependence (24). The selectivity for mutant over wild-type p110α likely reflects the greater conformational exposure of ubiquitination sites in mutants and preferential ligand binding, resulting in higher degradation efficiency in mutant cells (9).

### 2.3 Degradation efficiency and selectivity

In preclinical cellular models, inavolisib treatment reduced mutant p110α protein levels by ~80–90% within 24 hours, whereas decreases in wild-type p110α were generally modest (~15–25%), illustrating preferential degradation of mutant protein (9). This selectivity reflects greater conformational flexibility and solvent exposure of ubiquitination sites in mutant p110α, promoting ubiquitin conjugation and 26S proteasome–mediated turnover (9, 25, 26). The durability of pathway suppression in mutant models is notable: p-AKT and downstream signaling can remain suppressed for >72 hours after a single treatment in some studies, consistent with prolonged antiproliferative effects (27). In the Phase III INAVO120 study, addition of inavolisib to palbociclib and fulvestrant significantly extended median progression-free survival to 15.0 months versus 7.3 months (HR = 0.43) and improved ORR, providing clinical validation of the preclinical mechanism (28, 29). This drug overcomes the narrow therapeutic window limitation of traditional PI3K inhibitors by selectively degrading mutant p110α, providing a new strategy to overcome the reactivation of the insulin feedback pathway, which often leads to hyperglycemia and resistance in non-selective inhibitors (27).

## 3 The biological basis of combination therapy

**Figure 2:**
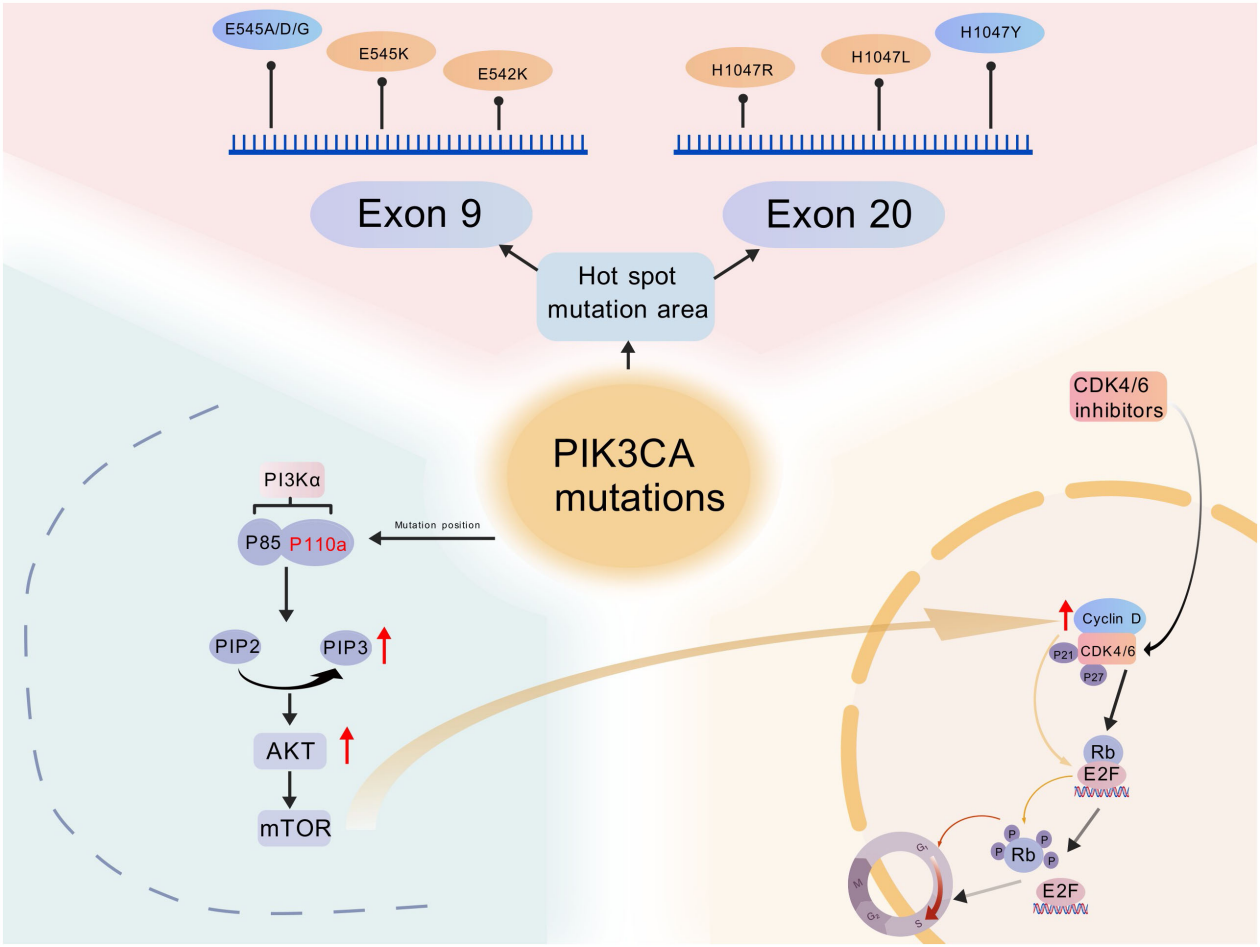
Schematic representation of PIK3CA mutations and their interaction with the PI3K/AKT/mTOR and CDK4/6-cyclin D-Rb pathways in breast cancer. (Created with BioGDP.com). Mutations lead to sustained activation of the PI3K/AKT/mTOR pathway, promoting tumorigenesis. CDK4/6 inhibitors block the CDK4/6-cyclin D-Rb-E2F axis, causing cell cycle arrest in the G1/S phase. Interactions between the overactive PI3K signaling pathway and the CDK4/6 pathway result in resistance. Rb, retinoblastoma protein.

Increasing evidence suggests that abnormal activation of the PI3Kα pathway, particularly mutations in the PIK3CA gene encoding the catalytic subunit of PI3Kα, is one of the key mechanisms mediating resistance to CDK4/6 inhibitors. PIK3CA mutations are frequently observed in various solid tumors. compensatory activation of CDK2/Cyclin E or mitogenic pathways (e.g., RAS/RAF/MEK/ERK) that restore Sphase entry despite CDK4/6 inhibition (30–35) (**Figure 2**).

### 3.1 Relationship between the PI3Kα pathway and PIK3CA mutations

PI3Kα is a heterodimer composed of the catalytic subunit p110α (encoded by the PIK3CA gene) and the regulatory subunit p85. Under the stimulation of growth factors such as EGF and IGF-1, it is activated through receptor tyrosine kinases (RTKs) or the RAS pathway, catalyzing the conversion of PIP2 to PIP3, which in turn activates the Akt/mTOR signaling axis, regulating cell proliferation, survival, and metabolism (36, 37).

PIK3CA mutations cluster in the helical (exon 9) and kinase (exon 20) domains; hotspot substitutions such as E542K, E545K, and H1047R account for ~70–75% of clinically observed variants (38, 39). Mechanistically, helical domain mutations (e.g., E542K/E545K) disrupt autoinhibitory interactions with the regulatory subunit p85, releasing catalytic activity, whereas kinase domain mutations (e.g., H1047R) enhance membrane interaction and substrate accessibility of p110α, increasing PIP3 production (40–42). These distinct mechanisms contribute to constitutive PI3K pathway activation and have implications for both inhibitor binding and degradation susceptibility (43).

PI3P abnormal accumulation leads to complete activation of Akt through phosphorylation at T308 site (mediated by PDK1) and S473 site (mediated by mTORC2) (36). Activated Akt promotes G1/S transition primarily by increasing Cyclin D1 expression and stability and by enhancing CDK4/6 activity, which leads to phosphorylation and functional inactivation of Rb, thereby liberating E2F transcription factors that drive S-phase gene expression (37, 44), and promoting ribosomal biosynthesis and energy metabolism by activating mTORC1, thereby providing the biomolecules required for tumor cell proliferation (45). Concurrently, Akt phosphorylation promotes the phosphorylation of pro-apoptotic proteins Bad (Ser136) and FoxO (e.g., FoxO1/3), causing them to bind to proteins and remain in the cytoplasm, thereby losing their pro-apoptotic function (46, 47).

### 3.2 Relationship between CDK4/6 inhibitor resistance mechanisms and PIK3CA mutations

Core mechanisms of resistance to CDK4/6 inhibitors include loss or functional inactivation of RB1. CDK4/6 inhibitors arrest the cell cycle by preventing phosphorylation of the Rb protein; loss of RB1 (e.g., homozygous deletion or inactivating mutations) or alternative post-translational modifications that negate Rb’s growth-suppressive function render cells intrinsically or acquiredly resistant to CDK4/6 blockade (30, 48). Parallel mechanisms include activation of the PI3K/AKT/mTOR pathway (via PIK3CA mutations or PTEN loss), which can upregulate Cyclin D1 and other cell-cycle drivers to bypass CDK4/6 dependence, and compensatory activation of CDK2/Cyclin E or mitogenic pathways (e.g., RAS/RAF/MEK/ERK) that restore S-phase entry despite CDK4/6 inhibition (30–35), render cells intrinsically or acquiredly resistant to CDK4/6 blockade (30, 48).

The molecular mechanism by which PIK3CA mutations impair the efficacy of CDK4/6 inhibitors involves several key processes. Mutations can directly interfere with the CDK4/6-Cyclin D-Rb pathway, activating the PI3K/AKT/mTOR pathway, inducing Cyclin D1 overexpression and enhancing its stability, while disrupting the regulation of cyclin-dependent kinase inhibitors (CKIs) such as p21 and p27, thereby counteracting the effects of CDK4/6 inhibitors (49). Activation of bypass signaling pathways, PIK3CA mutations, can activate the RAS/RAF/MEK/ERK pathway, promoting MYC-driven cell cycle escape (35). Preclinical studies have shown that the combination of PI3K inhibitors with CDK4/6i can reverse resistance, suggesting that bypass activation is a key mechanism underlying PIK3CA mutation-associated resistance (31, 50). Reprogramming in pathways such as glycolysis and lipid synthesis, supporting the rapid proliferation of drug-resistant cells (51). Concurrently, the upregulation of anti-apoptotic proteins (e.g., MCL-1) enhances cellular survival capacity, further reducing sensitivity to CDK4/6 inhibitors and promoting the survival of resistant clones (31).

### 3.3 Overcoming drug resistance

In cancer treatment, drug resistance is a challenging problem. Clinical studies have shown that when CDK4/6 inhibitors are used alone, some tumor cells can evade the inhibitory effects of the drugs by activating the PI3K/AKT/mTOR pathway, leading to the development of drug resistance. The emergence of Inavolisib offers new hope for addressing this issue. It achieves this by degrading mutated p110α, thereby fundamentally reducing the reactivation of the PI3K pathway. This allows CDK4/6 inhibitors to continue inhibiting tumor cell proliferation and maintain long-term therapeutic efficacy (9). Additionally, some tumor cells may bypass CDK4/6 inhibition by upregulating the Cyclin E-CDK2 complex, which is another important mechanism underlying tumor cell resistance. Inavolisib also plays a crucial role in this process. Inhibiting the PI3K pathway reduces Cyclin E expression and may indirectly suppress CDK2 activity through alternative signaling pathways, thereby successfully blocking this escape route and further enhancing the ability of combination therapy to overcome resistance (10, 52).

## 4 Synergistic mechanism of inavolisib with CDK4/6 inhibitors

**Figure 3:**
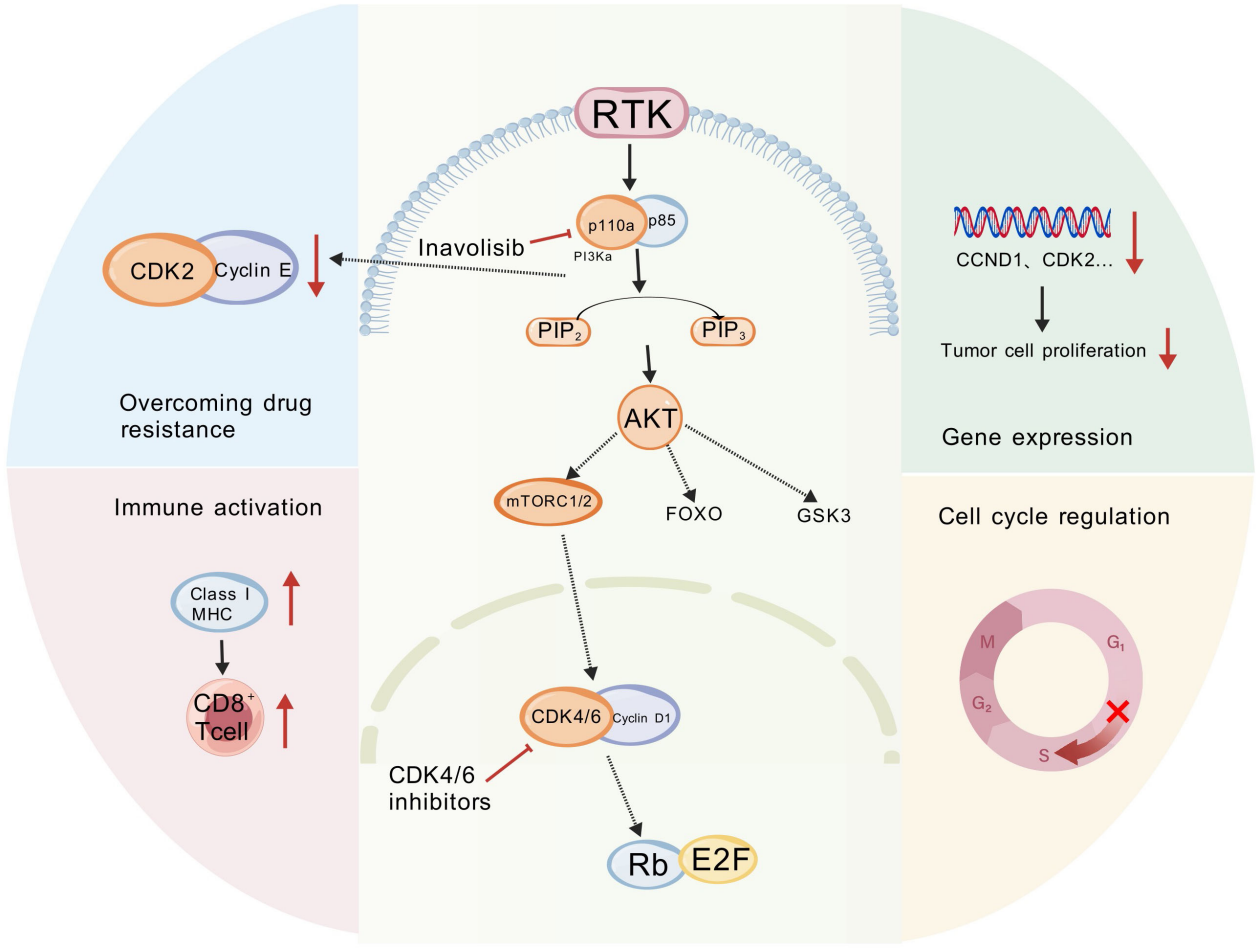
Synergistic mechanism of inavolisib with CDK4/6 inhibitors. (Created with BioGDP.com). The multidimensional impact of Inavolisib in combination with CDK4/6 inhibitors on tumor regulation. This figure illustrates a synergistic network where the combination therapy exerts antitumor effects by modulating key cellular processes, including drug resistance, immunity, gene expression, and cell cycle regulation. GSK3, Glycogen Synthase Kinase 3; FOXO, Forkhead Box O; CDK, Cyclin-Dependent Kinase; MHC, Major Histocompatibility Complex.

Combined inavolisib and CDK4/6 inhibition targets complementary tumor vulnerabilities—sustained PI3K signaling and aberrant cell-cycle progression—thereby addressing key resistance nodes and modulating the tumor microenvironment (**Figure 3**). Below, we summarize mechanistic evidence for synergy at signaling, cell-cycle, metabolic, and immune levels.

### 4.1 Blocking complementary signaling pathways

Inavolisib selectively inhibits and degrades mutant p110α, suppressing PI3K/AKT/mTOR signaling and reducing Cyclin D1 expression (28, 29). CDK4/6 inhibitors directly block Cyclin D–CDK4/6 kinase activity, preventing Rb phosphorylation and arresting cells in G1 (53–55). The combination therefore produces both upstream suppression of mitogenic signaling and downstream enforcement of cell-cycle arrest: inavolisib reduces drivers of Cyclin D expression while CDK4/6 inhibitors prevent residual CDK activity from phosphorylating Rb, producing a deeper and more durable block of G1→S progression (10, 55).

### 4.2 Synergistic effects at the molecular level

Multiple preclinical studies demonstrate that the combination yields deeper suppression of cell-cycle gene expression (e.g., CCND1) and key signaling readouts (p-RB, p-AKT, p-S6) than either agent alone. In models with ESR1 co-mutations, the combination prevented rebound of cell-cycle gene expression after initial suppression, suggesting sustained pathway blockade (10). At the protein level, selective degradation of mutant p110α by inavolisib eliminates primary oncogenic drivers, while CDK4/6 inhibitors constrain proliferation of residual cells, resulting in additive or synergistic antiproliferative effects (9).

### 4.3 Tumor microenvironment regulation

The tumor microenvironment strongly influences tumor progression and therapy response. Preclinical studies indicate that CDK4/6 inhibition can increase tumor cell MHC class I expression and augment T-cell activation, while also reducing regulatory T-cell infiltration in certain models, thereby enhancing cytotoxic T-cell–mediated immunity (56–58). Inavolisib, by blocking PI3K signaling, can alter tumor cell metabolism—reducing glycolysis and anabolic programs—and thereby limit nutrient utilization by tumor cells (59, 60). Evidence that CDK4/6 inhibitors reduce angiogenesis is model-dependent, but suppression of E2F-dependent pro-angiogenic programs, including VEGF, has been reported in some studies and may contribute to decreased tumor vascularization (55, 61). Collectively, these effects suggest that the combination can both restrict intrinsic tumor growth programs and remodel the immune and metabolic microenvironment to favor antitumor responses.

## 5 Synergistic antitumor effects in *in vitro*/*in vivo* models

Inavolisib combined with CDK4/6 inhibitors demonstrates a significant synergistic effect in a PIK3CA-mutated breast cancer model: *in vitro* experiments show an 89% inhibition rate in mutant cells (CI = 0.45), a 68% reduction in tumor volume *in vivo* models, and enhanced radiotherapy sensitivity while reshaping the tumor microenvironment (62).

### 5.1 *In vitro* experiments: synergistic proliferation inhibition and apoptosis induction of the combination of two drugs

*In vitro* experiments using appropriately characterized parental and engineered isogenic sublines demonstrated synergistic proliferation inhibition with the combination. Notably, studies that compared isogenic PIK3CA-mutant sublines (e.g., MCF-7/H1047R, T47D/E545K) to parental or corrected lines reported stronger synergy in mutant backgrounds (combination index CI ≈ 0.45 in mutant cells versus CI ≈ 0.58 in parental/corrected contexts), with inhibition rates in mutant models approaching 89%. It should be noted that commonly used parental MCF-7 and T47D lines frequently carry heterozygous PIK3CA hotspot mutations (E545K in T47D; E545K or H1047R in MCF-7, depending on the subline), so explicit reporting of genotype/isogenic status is essential when interpreting results. This is closely related to the characteristic of PIK3CA mutations leading to enhanced dependence on the PI3K/AKT/mTOR pathway (5, 6). Mechanistic studies revealed that the combination therapy induces deep G1 phase arrest (G1 phase proportion increased from 65% to 82%) and apoptosis activation (caspase-3 activation increased threefold) by dual inhibition of RB phosphorylation and AKT/mTORC1 signaling, while downregulating Cyclin D1 (reduced by 75%) and EMT marker Vimentin (decreased by 50%) (63). Metabolic analysis showed that the combination regimen reduced ECAR by 55% and increased OCR by 20%, suggesting a shift in metabolic reprogramming toward oxidative phosphorylation, consistent with its inhibition of mTORC1 (p-S6K1 decreased by 70%) and activation of AMPK (Thr172 phosphorylation increased twofold) (64, 65).

### 5.2 *In vivo* models: tumor growth inhibition and mechanism validation of combination therapy

Multiple PDX and cell-line xenograft studies validate the synergistic antitumor activity of combined PI3Kα and CDK4/6 inhibition; some models showed durable regressions while others exhibited tumor stasis, highlighting dependency on tumor genotype and treatment scheduling (52). In PIK3CA-mutant xenografts (e.g., engineered MCF-7/H1047R models), combination therapy produced marked tumor growth inhibition and extended tumor doubling times relative to monotherapy (8, 66, 67). Additionally, in immune-reconstituted xenograft models, combined PI3Kα and CDK4/6 inhibition has been associated with remodeling of the tumor immune microenvironment, including increased CD8+ T cell infiltration and enhanced cytotoxic effector markers (such as Granzyme B) (56, 68, 69). These findings suggest that dual targeting may not only arrest tumor cell proliferation but also augment antitumor immunity; however, inter-study variability and dependence on model immunocompetence require cautious interpretation and further validation.

### 5.3 Radiation sensitization potential: CDK4/6 inhibitors combined with Inavolisib enhance the efficacy of radiotherapy

Preclinical studies show that inhibition of PI3K signaling or CDK4/6 can sensitize tumor cells to ionizing radiation by impairing DNA damage repair processes and altering cell-cycle distribution, thereby increasing persistent DNA damage and radiosensitivity (70, 71). This mechanistic rationale supports preclinical evaluation of radiosensitizing effects for inavolisib-containing combinations (72–74). However, direct, robust evidence specifically for inavolisib plus CDK4/6 inhibitors in clinically relevant models is limited, and additional focused studies are required before translation to clinical trials (9, 68, 75).

## 6 Clinical research progress

Clinical data increasingly support the translational relevance of the preclinical rationale. In the Phase III INAVO120 study, addition of inavolisib to palbociclib and fulvestrant significantly extended median progression-free survival to 15.0 months versus 7.3 months in the control arm (hazard ratio 0.43) and increased objective response rate (ORR) to 58.4% compared with ~25% in the control arm, demonstrating meaningful clinical benefit in PIK3CA-mutated HR+/HER2- advanced breast cancer (28, 29). Earlier phase I/II studies (e.g., NCT03386149) established a recommended phase II dose of inavolisib (160 mg QD) and suggested a favorable metabolic safety profile relative to first-generation PI3K inhibitors, with lower rates of high-grade hyperglycemia in these cohorts (18, 68).Regarding safety, the INAVO120 study reported an increased incidence of hematologic toxicity consistent with CDK4/6 inhibition (notably neutropenia), but rates of metabolic toxicities typically associated with PI3K inhibition, including high-grade hyperglycemia, were comparatively low with inavolisib in this program (grade ≥3 hyperglycemia ~5-7% in reported cohorts) (28, 29, 76). Treatment discontinuation due to adverse events was modest in trial reports (~6–9%), suggesting the combination can be administered with manageable toxicity using standard supportive measures and dose modifications (68).

In a combination therapy strategy, the MORPHEUS-pan BC Ib/II phase study demonstrated that the combination of Inavolisib with Abemaciclib or Ribociclib did not significantly increase the risk of high-grade toxicity, potentially due to Inavolisib’s selective degradation of mutant p110α protein (9, 18). Compared with the median PFS of 7.2 months and ORR of 26.6% observed in the BYLieve study with Alpelisib plus fulvestrant (77, 78), the INAVO120 Phase III study demonstrated that Inavolisib in combination with palbociclib and fulvestrant extended median PFS to 15.0 months (HR = 0.43, p<0.001), and ORR improved to 58.4% (79). The mechanism underlying the improved efficacy may be attributed to Inavolisib’s specific degradation of mutated p110α protein, which blocks PI3K/AKT/mTOR signaling and reduces bypass activation, whereas Alpelisib only inhibits enzyme activity, leading to residual signaling and resistance (9, 18). In terms of safety, the incidence of grade ≥3 hyperglycemia in the INAVO120 study was only 5.6%, significantly lower than the 37% reported in the BYLieve study (68, 79), and the incidence of rash (18% vs 54%) and diarrhea (22% vs 45%) was also significantly reduced, indicating that Inavolisib reduces non-specific inhibition of wild-type PI3Kα (18). Inavolisib in combination with CDK4/6 inhibitors achieves dual blockade of PI3Kα and the cell cycle, significantly improving PFS and OS (median PFS extended to 15.0 months) in HR+/HER2- PIK3CA-mutated patients (28).

## 7 Key issues in optimizing treatment strategies

Optimizing treatment strategies with inavolisib and CDK4/6 inhibitors requires addressing several key issues. First, the precise identification of the patient population who will benefit is critical, which relies on the accurate detection of PIK3CA mutations. Second, monitoring for and overcoming drug resistance through methods like dynamic ctDNA analysis is essential for long-term efficacy. Finally, managing the toxicities associated with combination therapy, such as myelosuppression and hepatotoxicity, is crucial to balance treatment efficacy and patient safety.

### 7.1 Patient selection and biomarkers

#### 7.1.1 Clinical significance of PIK3CA mutation detection

PIK3CA mutations occur in roughly 30–50% of HR+/HER2- breast cancers, though exact frequencies vary with cohort composition and detection methods (14, 32, 80). Hotspot substitutions such as H1047R, E545K, and E542K comprise the majority of clinically relevant variants. Importantly, both the presence and the allele fraction of PIK3CA mutations (in tissue or ctDNA) influence sensitivity to PI3K-targeted therapy and should guide patient selection for inavolisib-based combinations. PIK3CA mutations lead to sustained activation of the PI3Kα pathway, promoting tumor proliferation and invasion (32). Clinical studies have confirmed that patients with PIK3CA mutations are more sensitive to PI3Kα inhibitors (such as Inavolisib). For example, the median progression-free survival (PFS) was significantly prolonged in patients with PIK3CA mutations who received Inavolisib in combination with endocrine therapy or CDK4/6 inhibitors compared to non-mutated patients (28). A meta-analysis further confirmed that PIK3CA mutations are an independent predictive factor for poor prognosis (shortened PFS and OS) in HR+/HER2- breast cancer patients (31). PIK3CA mutation testing is crucial for precision screening of treatment-benefiting populations, avoiding unnecessary toxicity and improving efficacy (28, 32).

#### 7.1.2 Drug resistance monitoring and ctDNA dynamic analysis

ctDNA monitoring enables longitudinal surveillance for emergent resistance alterations during targeted therapy. In PI3K-targeted programs, rising PIK3CA allele fraction, acquisition of secondary PIK3CA mutations, or detection of bypass events (e.g., PTEN loss, RTK amplifications) may precede radiographic progression and can guide timely therapeutic modifications (81–83). Incorporating standardized ctDNA assays into trials of inavolisib combinations will improve early detection of resistance and facilitate rational sequencing or addition of agents. In HER2-positive breast cancer, ctDNA detection of HER2 amplification or PIK3CA/MTOR pathway mutations significantly increases the risk of resistance (sensitivity 85.7%, specificity 55%) (81). Through dynamic ctDNA monitoring, clinicians can promptly adjust treatment regimens (e.g., switch drugs or combine with other targeted therapies) and provide direction for resistance mechanism research (82, 83).

### 7.2 Adverse reaction management

Combination therapy carries overlapping toxicities—most commonly hematologic suppression (neutropenia related to CDK4/6 inhibitors) and metabolic or hepatic events associated with PI3K inhibition (28, 84). Management strategies include baseline risk assessment, regular laboratory monitoring (including weekly blood counts during initiation and frequent ALT/AST checks), dose interruptions/reductions, and supportive care (e.g., G-CSF for prolonged Grade ≥3 neutropenia) (85). Hyperglycemia should be monitored and treated per standard practice (dietary measures and oral hypoglycemics such as metformin when indicated) (28, 76). Potential drug-drug interactions (e.g., CYP3A4 modulators) should be reviewed to reduce hepatic risk (84). Sequential therapy may mitigate overlapping toxicities but could permit tumor rebound (86, 87). Therefore, individualization of the schedule and close monitoring are essential (28, 86).

## 8 Future directions and challenges

### 8.1 Exploration of new combination therapies

Exploring new combination therapies, particularly with immunotherapy, represents a promising future direction. Combining PI3K pathway inhibitors with immune checkpoint blockade is mechanistically attractive: PI3K modulation can remodel tumor-associated myeloid cells, reduce immunosuppressive cell populations, and augment T-cell effector function, thereby potentiating responses to PD-1-1/PD-PD-L1 blockade in some preclinical models. For example, after inhibiting the PI3Kγ pathway, Copanlisib increases the infiltration of CD8+ T cells in the tumor microenvironment, reduces Treg cells, and elevates levels of pro-inflammatory cytokines such as IFN-γ (88, 89). Preclinical studies have shown that the combination of PI3K inhibitors with PD-1/PD-L1 inhibitors significantly extends the survival of tumor-bearing mice, suggesting that this strategy may overcome resistance to immunotherapy (90). Given inavolisib’s selectivity for mutant p110α and the immunomodulatory effects of CDK4/6 inhibitors, rational triplet combinations (inavolisib + CDK4/6 inhibitor + immune checkpoint inhibitor, ICI) merit preclinical evaluation with careful attention to dosing and immune-related toxicity.

### 8.2 Translational medicine research

Drug resistance limits the durability of targeted combinations. Integrative translational studies that combine serial tumor and ctDNA genomics with proteomic and functional pharmacology (including CRISPR/RNAi screens and phosphoproteomics) are needed to delineate resistance trajectories and to prioritize co-targets. Candidate avenues include inhibition of CDK2/Cyclin E, mTORC2, or components of the translational machinery (e.g., eIF4E) to counteract adaptive reactivation of growth signaling and protein synthesis that underlie resistance (31, 50). For example, excessive activation of eIF4E (a translation initiation factor) can promote the synthesis of resistant proteins (31). Such data should inform biomarker-driven, adaptive clinical trial designs (31, 91).

## 9 Conclusion

In PIK3CA-mutated HR+/HER2- breast cancer, targeted degradation and inhibition of mutant p110α with inavolisib combined with CDK4/6 inhibition offers a compelling strategy to block both oncogenic signaling and cell-cycle progression. Preclinical and Phase I–III data demonstrate enhanced efficacy and manageable toxicity when appropriate selection and monitoring strategies are used. To maximize the clinical benefit, continued translational research is required to refine biomarkers (including ctDNA dynamics and co-mutation profiles), optimize dosing and scheduling to minimize overlapping toxicity, and to develop rational triplet or sequential strategies to prevent or overcome resistance. Furthermore, understanding how this combination therapy remodels the tumor immune microenvironment will be key to developing novel strategies, potentially involving immunotherapy, to further improve patient outcomes.
